# Dual triggering for final oocyte maturation. A narrative review

**DOI:** 10.3389/fendo.2025.1556732

**Published:** 2025-05-22

**Authors:** Adriana Riobó, Andrea Martínez Acosta, Lorena Martinez-Rocca, Esther Taboas, Belén López de Uralde, Iria Fernandez, Nicolás Garrido, Elkin Muñoz

**Affiliations:** ^1^ IVIRMA GLOBAL Research Alliance, IVI Coruña, A Coruña, Spain; ^2^ IVIRMA GLOBAL Research Alliance, IVI Vigo, Vigo, Spain; ^3^ IVIRMA GLOBAL Research Alliance, IVI Foundation – Instituto de Investigación Sanitaria La Fe (IISLa Fe), Valencia, Spain; ^4^ Department of Obstetrics and Gynecology, University of Cauca, Popayan, Colombia

**Keywords:** dual triggering, final oocyte maturation, GnRH agonists, hCG, low ovarian responders, immature oocytes retrieved, clinical pregnancy rate, live birth rate

## Abstract

The dual triggering combines human chorionic gonadotropin (hCG) with GnRH agonists (aGnRH) to induce the final oocyte maturation in *in vitro* fertilization (IVF). When both drugs are used sequentially, it is called “double trigger”, but this strategy is rarely used clinically. This review explores the rationale for using dual triggering and compares its reproductive outcome with conventional hCG triggering. Variability in protocols, inclusion criteria, study aims, and weak study designs complicate the evaluation of its clinical benefit. Patients with low response or cycles with high proportion of immature oocytes (>25%) may benefit from dual triggering. In contrast, patients with normal or hyper responsiveness show no significant differences compared to conventional hCG triggering. Further robust studies are needed to clarify the clinical applications of dual triggering. Until then, this strategy should remain part of research protocols rather than routine clinical practice.

## Introduction

Final oocyte maturation is naturally triggered by the pre-ovulatory surge of luteinizing hormone (LH). This process is crucial for both natural conception and assisted reproductive techniques (ART). LH induces oocyte maturation, promotes ovulation and the transformation of the follicle into the corpus luteum, which support early pregnancy development. The production of progesterone by the corpus luteum induces the transformation of the proliferative endometrium into secretory one, allowing embryo implantation (1).

The follicle is the functional unit of the ovary, playing key roles in both reproductive and endocrine functions (2). Ovarian follicle growth occurs in two stages: the first, from the primordial to the pre-antral follicle, which is gonadotropin-independent, and the second, from the pre-antral to the ovulatory follicle, which is gonadotropin-dependent (3). Follicle-stimulating hormone (FSH) initiates follicular development, making this first stage FSH-dependent, while luteinizing hormone (LH) promotes androgen secretion by the theca cells and is involved in follicular dominance, complete maturation, ovulation, and the support of the corpus luteum (4, 5). The exogenous administration of FSH increases the number of FSH receptors in granulosa cells, enhancing their sensitivity to FSH. This continuous stimulation surpasses the FSH threshold seen in the natural cycle, preventing the selection of a dominant follicle. As a result, multiple follicles grow under the effect of ovarian stimulation for *in vitro* fertilization (IVF) (6).

The mid-cycle surge of LH induces changes in the dominant follicle culminating in the ovulation and transformation of the ruptured follicle into corpus luteum (7).

Due to the difficulties for synthesizing LH in an amount equivalent to naturally released with the capacity of inducing final oocyte maturation (8), drugs with LH-like activity, such as human chorionic gonadotropin (hCG), as well as drugs that induce the endogenous release of LH, such as gonadotropin-releasing hormone agonists, are widely used in assisted reproduction treatments (1).

Oocytes remain immature (germinal vesicle arrested in prophase I or in metaphase I) until the pre-ovulatory LH surge. Final oocyte maturation is a critical step, but not all stimulated oocytes mature properly or reach the mature stage at the same time. In assisted reproduction cycles, in which more than 25% of immature oocytes are retrieved, fertilization rates and clinical pregnancy are reduced drastically (9). With the induction of oocyte maturation, it is expected to obtain between 75-85% of mature oocytes (10). If a program of oocyte *in-vitro* maturation is not available, these immature oocytes are usually discarded (10).

hCG was the first trigger and it is the most widely used. It is an effective inducer for triggering oocyte maturation, with exclusively LH activity. This suggests that the elevation of follicle stimulating hormone (FSH) in the middle of the natural cycle seems to play a secondary role in final oocyte maturation (9). On the other hand, GnRH agonists (aGnRH) promote the release of not only LH but also FSH that is responsible for amplifying LH activity, supporting the formation of LH receptors in granulosa cells, favoring cumulus expansion and nuclear maturation (11). Besides, the short duration of the LH surge induced by the GnRH agonist to trigger oocyte maturation could explain the notable reduction in the risk of ovarian hyperstimulation syndrome (OHSS) (12).

Recently, “dual triggering” has been used to describe the combination of hCG and aGnRH, which may synergically increase the number of mature oocytes. Its potential benefits have been studied in cases of low ovarian response, poor fertilization rate, suboptimal reproductive outcomes and fertility preservation (13).

A retrospective study included fresh embryo transfer cycles of 1068 women that underwent dual triggering and 1931 women that underwent hCG-only triggering from the Poseidon groups 3 and 4. Number of retrieved oocytes per cycle (4.11 vs 3.73), MII oocytes per cycle (3.3 vs 2.6), oocyte maturation rate per cycle (0.82 vs 0.73), fertilization rate per cycle (0.77 vs 0.72), obtained embryos per cycle (2.34 vs 1.72), implantation rate (24% vs 20%), clinical pregnancy rate per cycle (28.9% vs 25%) and live birth rates (LBR) per cycle (24.9% vs 18.2%) were found significantly higher in dual triggering group (*p* < 0.001 in each one respectively) (14). Similar results were described when 1010 low responder patients according to Bologna criteria were retrospectively analyzed in terms of final oocyte maturation with dual trigger compared with conventional hCG trigger. Fertilization rates (73.6% vs 69.6%) implantation rates (18.7% vs 14.6), clinical pregnancy rate per embryo transfer (27.5% vs. 19.9%), and live birth rate per embryo transfer (21.6% vs. 14.9%) were significantly higher in the dual trigger group (15).

Another recent systematic review included 1390 studies of which 7 studies were in 2474 low responders examined whether the dual trigger is beneficial or not with respect to the implantation, pregnancy and live birth rates. The meta-analysis revealed an increase in clinical pregnancy rate (OR = 1.62) and an increase in live birth rate (OR = 2.65) in the dual trigger group compared to hCG trigger. The pooled analysis showed no significant difference between the two groups regarding implantation rate (OR = 1.14) (16).

The results of dual trigger were evaluated in patients whose immature oocyte rate in the previous cycle was more than 50%. Thirty-nine patients with normal ovarian response using dual triggering were compared with 34 patients with hCG trigger. The primary outcome was oocyte maturation rate (proportion of MII oocytes from the total number of retrieved oocytes), and it was higher in dual trigger group (84.0% vs. 55.5%). The cumulative pregnancy rate (69.4% vs. 40.0%), and cumulative live birth rate (66.7% vs. 36.0%), were also higher in the dual trigger group (17).

The objective of this review is to describe the rationale of using dual triggering for final oocyte maturation in *in vitro* fertilization (IVF), and to compare the reproductive outcomes between dual triggering vs conventional hCG triggering.

## Physiological mechanisms

**Figure 1:**
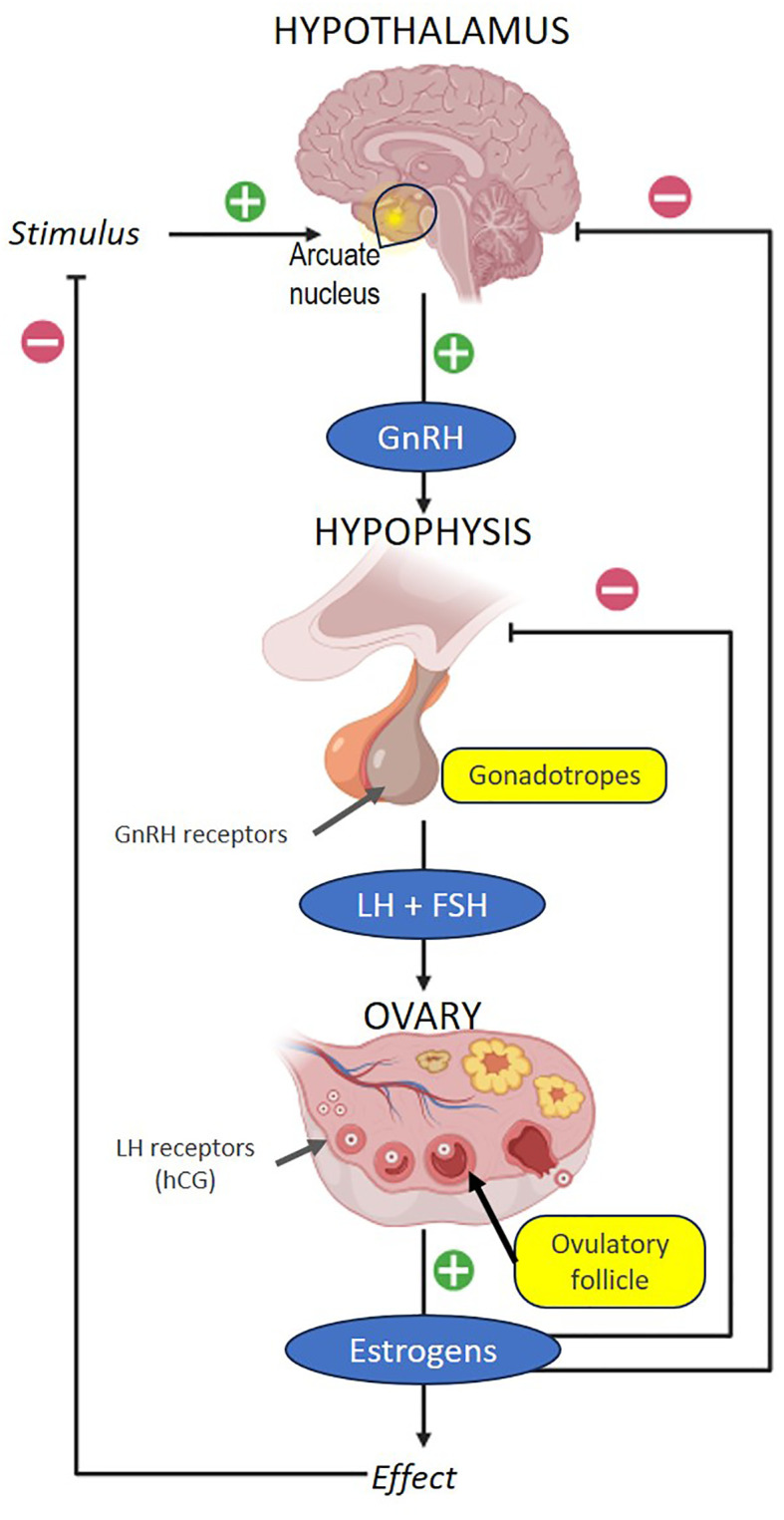
Neuroendocrine regulation of ovulation.

It is not entirely clear when oocyte maturation from metaphase I to metaphase II occurs at the second meiotic division, but it is thought to be related to the LH peak which could block the effect of oocyte maturation inhibitor (OMI). Ovulation occurs in response to an ordered sequence of events that begins with the increase in estradiol produced by the pre-ovulatory follicle, which begin to raise LH 34–36 hours before follicular rupture. The LH peak is reached 12 hours before ovulation and a classic study showed that it is necessary for this threshold level to be maintained for 14–27 hours to achieve oocyte maturation (18). Gonadotropins are released from gonadotropes cells in the anterior pituitary gland because of the pulsatile releasing of GnRH from the arcuate nucleus located at the base of the hypothalamus. These gonadotropins promote cumulus expansion, resumption of meiotic maturation, and follicle rupture. A summary of the endocrine regulation cascade of ovulation is outlined in **Figure 1**.

*In vivo* oocyte maturation is a complex process regulated by hormonal signals, cellular interactions, transcription and expression of regulatory genes. The molecules that regulate this process are produced by the granulosa cells, such as OMI, which depends on the integrity of the clusters due to gap junctions. With the LH surge, the cyclic AMP (cAMP) is transported from the granulosa cells to the oocyte, generating an increase in cAMP and rupture of the gap junctions of the clusters and the subsequent loss of OMI activity facilitating the resumption of meiosis. After the LH peak, the granulosa cells express a greater number of LH receptors, which induce the expression of growth factors such as endothelial growth factor (EGF)-Like, that block the gap junctions. Resuming meiosis culminates with the extrusion of the first polar body, obtaining a mature oocyte in metaphase of the second meiotic division (MII) (19, 20).

**Figure 2:**
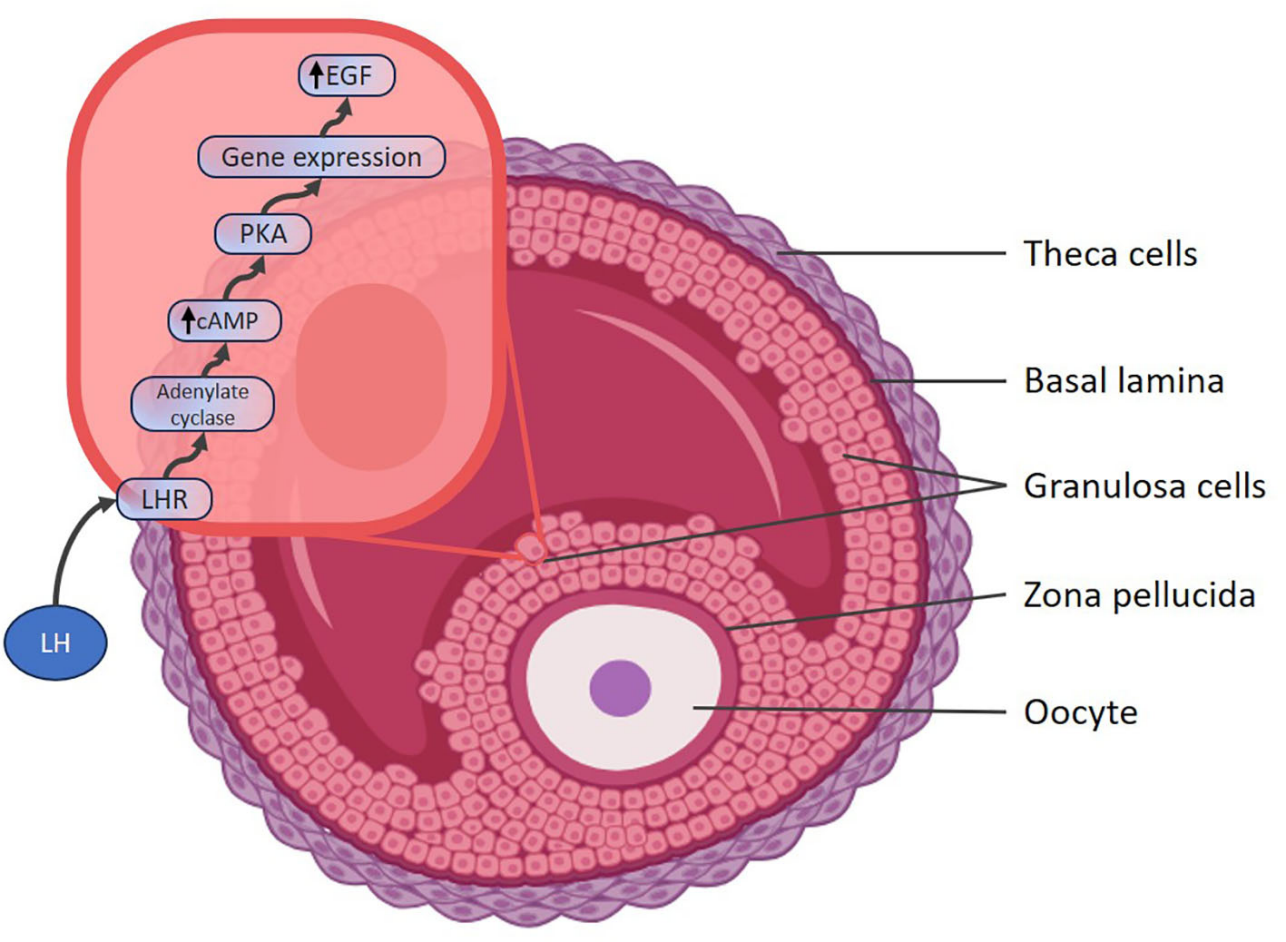
LH action on granulosa cells and cluster cells. LH, Luteinizing hormone; LHR, LH receptor; cAMP, cyclic adenosine monophosphate; PKA, Protein Kinase A; EGF, endothelial growth factor.

Oocyte maturation results from a complex process in which merely preventing exposure to oocyte maturation inhibitor (OMI) is insufficient to ensure oocyte maturation. Achieving the ovulation of a mature oocyte requires inputs received by the follicle, including hormonal, immune, and metabolic signals. Additionally, intrafollicular paracrine factors from theca cells, mural granulosa cells, cumulus granulosa cells, and the oocyte itself play a crucial role (19) (**Figure 2**).

An oocyte is considered mature after completing nuclear and cytoplasmic maturation. Nuclear maturation involves the resuming meiosis until the MII phase. This process begins with the resumption of meiosis that is signaled by the disappearance of the nuclear membrane of the germinal vesicle and its breakdown, subsequent condensation of chromatin, formation of polar spindles, separation of chromosomes and ends with the extrusion of the first polar corpuscle. Cytoplasmic maturation involves organelle reorganization, enabling oocyte activation. While nuclear maturation is visible through the extrusion of the first polar body, cytoplasmic maturation is harder to assess clinically, and it is inferred from the oocyte`s performance (e.g. normal fertilization and embryonic development). The second meiotic division will be completed with fertilization when the sperm enters the oocyte (21).

## Pharmacological properties

### hCG

hCG is indicated for the treatment of female infertility as a final ovulation induction therapy. Other indications of hCG includes male infertility (22) and fertility preservation (23). It may be a recombinant human chorionic gonadotropin (rhCG) obtained through genetic engineering techniques, with its active ingredient being choriogonadotropin alfa (24).

hCG is structurally similar to luteinizing hormone (LH), as both are glycoproteins sharing the same alpha subunit and 85% of the amino acid structure of the beta subunit. This similarity enables hCG to stimulate LH receptors, inducing luteinization of granulosa cells, resuming meiosis, and promoting ovulation (10). However, the binding of hCG to LH receptors differs slightly from that of LH. Structural variations allow hCG to have a greater affinity for LH receptors and a longer half-life over 24 hours compared to approximately 60 minutes for LH. This prolonged activity leads to sustained luteotrophic effects, including the release of vasoactive agents such as vascular endothelial growth factor (VEGF), thereby increasing the risk of ovarian hyperstimulation syndrome (OHSS) (8).

Choriogonadotropin alfa is a water-soluble glycoprotein composed of two non-covalently linked subunits—designated α and β—comprising 92 and 145 amino acid residues, respectively, with carbohydrate moieties linked to ASN-52 and ASN-78 (on the alpha subunit) and ASN-13, ASN-30, SER-121, SER-127, SER-132, and SER-138 (on the beta subunit). The primary structure of the α-chain of rhCG is identical to that of the α-chain of hCG, FSH, and LH. The glycoform pattern of the α-subunit of rhCG is closely comparable to urinary-derived hCG (u-hCG), with differences mainly in the branching and sialylation of the oligosaccharides. The β-chain has both O- and N-glycosylation sites, with a glycosylation pattern also similar to that of u-hCG (25).

The physicochemical, immunological, and biological activities of rhCG are comparable to those of placental and urine-derived hCG from pregnant women. Choriogonadotropin alfa acts like LH that binds to the LH/hCG receptor on granulosa and theca cells of the ovary, inducing these changes in the absence of an endogenous LH surge (26). During pregnancy, hCG secreted by the placenta maintains corpus luteum viability, ensuring continued secretion of estrogen and progesterone necessary to support the first trimester and prevent miscarriages (27).

Choriogonadotropin alfa binds to the LH receptor, inducing ovulation in the absence of sufficient endogenous LH. The principal pharmacodynamic activity in women includes resuming oocyte meiosis, follicular rupture (ovulation), corpus luteum formation, and the production of progesterone and estradiol, roles typically performed by the corpus luteum in natural cycles. Chorionic gonadotropin serves as a surrogate for the luteinizing hormone peak, triggering ovulation (28, 29).

The administration of choriogonadotropin alfa can be intramuscular or subcutaneous. The dosage regimen depends on the indication, age and weight of the patient, and the physician’s preference, but a subcutaneous dose of 6500 IU is the most common. Following administration, choriogonadotropin alfa is distributed into the extracellular fluid space with a distribution half-life of approximately 4.5 hours. The steady-state volume of distribution and total clearance are 6 L and 0.2 L/h, respectively (26). Circulating hCG is metabolized primarily in the liver, with approximately 20% excreted via the kidneys (30). The terminal half-life is about 30 hours, and the absolute bioavailability is around 40% (25).

### GnRH agonists

aGnRHs are derived from native GnRH through amino acid substitution, which makes the agonist resistant to degradation and increases its half-life. aGnRHs stimulate the pituitary gland to release both LH and FSH, causing a short but intense surge. This initial response is followed by downregulation and inhibition of the pituitary-gonadal axis, as the pituitary becomes less responsive to GnRH, leading to a decrease in LH and FSH production (31). By first stimulating gonadotropin release and then downregulating it, aGnRH can help normalize steroid hormone levels (32).

**Figure 3:**
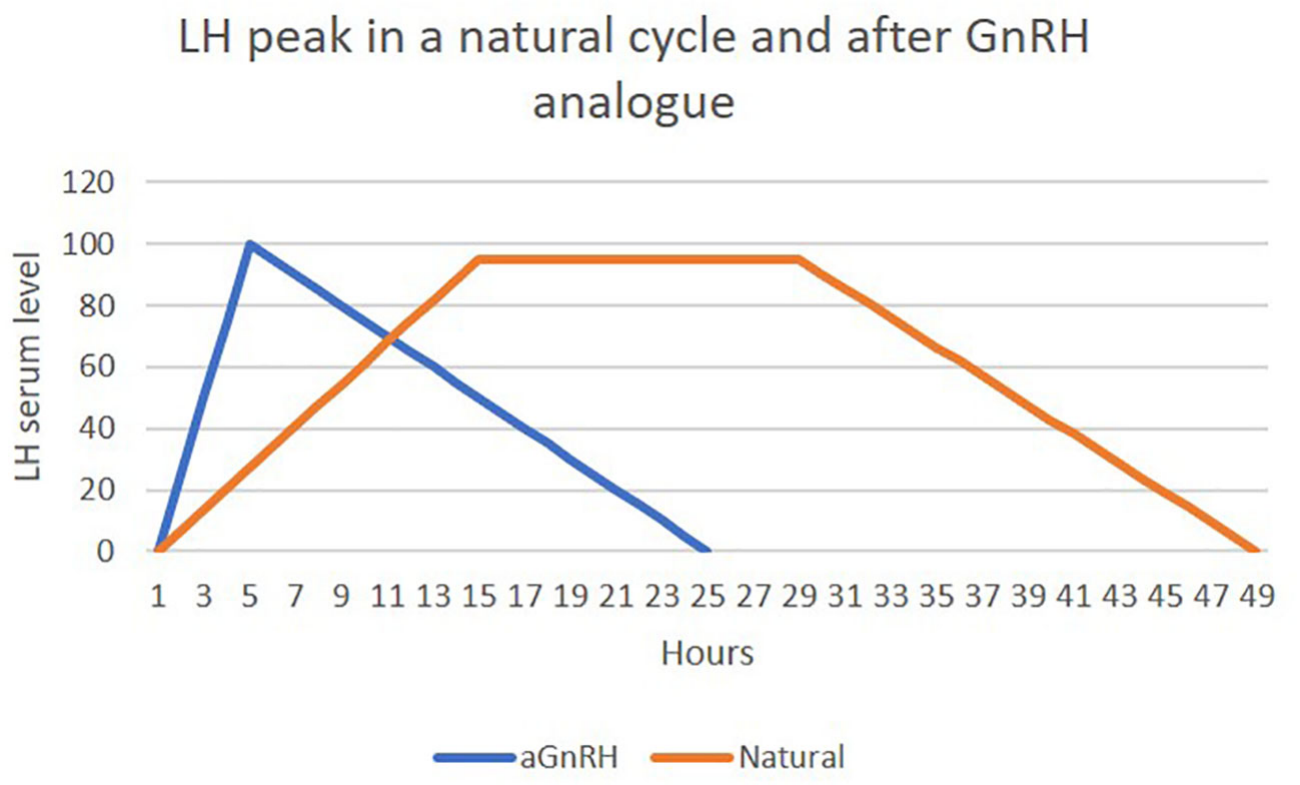
Differences in LH serum levels between natural cycle and after aGnRH. own creation- adapted from: Martinez B. et al. Luteal phase after GnRH agonist triggering ovulation. 11th International Symposium on GnRH: The Hypothalamic-Pituitary-Gonadal Axis in Cancer and Reproduction. 2014- Salzburg- Austria.

aGnRHs exert their effects at the pituitary level, meaning they can only be used in stimulation cycles with GnRH antagonists or without pituitary suppression. Their primary role is to occupy GnRH receptors in the pituitary, triggering the release of FSH and LH and generating an ovarian response known as the “flare-up” effect. This effect has two phases: a short ascending phase (4 hours) and a longer descending phase (20 hours), totaling 24–36 hours. LH induced by natural GnRH persists longer, acting through three phases over 48 hours. With aGnRHs, luteolysis occurs, causing a steroid deficit during the luteal phase, thereby almost completely preventing OHSS. If pregnancy is pursued in the same cycle, luteal phase rescue is necessary to prevent adverse effects on implantation and clinical pregnancy rates (33) (**Figure 3**).

Various aGnRH have been used in Europe, with triptorelin and buserelin being the most common. In the United States, nafarelin (800–1800 mcg/day intramuscularly) and leuprolide (4–50 mcg/kg/day subcutaneously) have been effective (34). Deslorelin is currently used to promote ovulation and treat high-risk pregnancies in animals (32). After an initial spike in GnRH-mediated steroid production (including testosterone and estradiol), prolonged use leads to a significant drop in circulating steroid levels, like other forms of androgen-deprivation therapy (35).

The elimination routes vary among specific drugs but generally involve both the kidneys and the liver (36).

Higher doses administered subcutaneously achieve effects comparable to intravenous and intramuscular administration. However, subcutaneous administration results in smaller blood peaks that develop more slowly and take longer to return to baseline. Other administration methods include nasal sprays, sustained-release implants, and injections of biodegradable microspheres (32, 37).

Metabolism also varies among synthetic agonists. The metabolism of triptorelin likely does not involve hepatic enzymes such as cytochrome P450, and its effect on other metabolizing enzymes is poorly understood. Triptorelin has no identified metabolites, while nafarelin undergoes enzymatic hydrolysis (37).

### Dual triggering

The simultaneous use of hCG and aGnRH for final oocyte maturation is referred to as “dual triggering.” When these drugs are administered sequentially, this is known as the “double triggering” protocol. In the latter case, GnRH agonist and recombinant hCG are administered 40 hours and 34 hours before oocyte retrieval, respectively. However, this approach is less clinically accepted and poorly studied (29).

The action of hCG/LH plays a dominant role in final oocyte maturation. However, the addition of FSH provided by aGnRH warrants further study, as it may increase the number of mature oocytes or facilitate cytoplasmic maturation of the oocyte.

## Clinical results in dual triggering *vs.* standard protocol

### Low ovarian response

Among different patient profiles, those with low ovarian response (LOR) as defined by the Bologna and/or Poseidon criteria have been the most extensively studied. In 2011, the European Society of Human Reproduction and Embryology (ESHRE) introduced the Bologna criteria for LOR, and later in 2016, the Poseidon criteria were established to classify patients more homogeneously. Among women aged 35–40 years, 10–15% are poor responders (38). In this patient group, alternative methods of inducing final oocyte maturation are sought to achieve a greater number of retrieved oocytes (39).

Studies on patients with LOR (e.g., Bologna or Poseidon criteria) consistently demonstrate improved outcomes with dual triggering compared to hCG alone.

In the first randomized controlled trial (RCT), patients underwent unblinded randomization after completing an initial cycle with LOR. The study compared the efficacy of dual triggering (10,000 IU of hCG plus 0.2 mg of triptorelin) versus a single hCG trigger (10,000 IU). A total of 160 patients with LOR, defined by the Bologna criteria and undergoing intracytoplasmic sperm injection (ICSI), were randomized into two groups of 80 each via an automated web-based randomization system. Results showed significantly higher numbers of retrieved oocytes (5.3 ± 1.9 vs. 4.5 ± 2.4, *p* = 0.014), mature oocytes (3.8 ± 1.4 vs. 3.1 ± 1.7, *p* = 0.004), total embryos (2.7 ± 1.1 vs. 1.9 ± 1.2, *p* = 0.001), and good-quality embryos (2.3 ± 1.0 vs. 1.1 ± 0.2, *p* = 0.021). Additionally, the clinical pregnancy rate per ovarian stimulation cycle was higher in the dual triggering group (22.5% vs. 8.8%, *p* = 0.028) (40). However, a major limitation of this study was the inability to calculate ongoing pregnancy and live birth rates due to significant patient loss during follow-up.

A retrospective cohort study comparing dual triggering to hCG alone analyzed 384 patients with LOR and Poseidon 4 criteria (women >35 years old, with AFC < 5 and/or AMH < 1.2 ng/mL). It included 114 patients receiving hCG alone and 194 receiving dual triggering. The dual triggering group showed significantly higher numbers of retrieved oocytes (3.3 ± 2.7 vs. 1.6 ± 1.5, *p* < 0.001), metaphase II oocytes (2.6 ± 2.0 vs. 1.3 ± 1.0, *p* < 0.001), fertilized oocytes (2.4 ± 2.1 vs. 1.2 ± 1.0, *p* < 0.001), day-3 embryos (2.2 ± 1.9 vs. 1.2 ± 1.0, *p* < 0.001), and top-quality embryos (0.9 ± 1.3 vs. 0.2 ± 0.5*, p* < 0.001). Clinical pregnancy rates (23.1% vs. 8.7%, *p* = 0.004) and live birth rates (17.5% vs. 5.4%, *p* = 0.006) were also significantly improved in the dual triggering group (41). Fertilization rates, however, did not differ between groups (74.3 ± 37.4 vs. 77.8 ± 39.4).

Another comparative study involving patients under 35 years of age with diminished ovarian reserve showed improvements in fertilization rates (73.1% vs. 58.6%, *p* = 0.015) and live birth rates (27.2% vs. 13.1%, *p* = 0.014) with dual triggering. The dual triggering group also had a lower cycle cancellation rate (6.1% vs. 15.4%, *p* < 0.003), though no significant differences were observed in the number of retrieved oocytes, mature oocytes, or embryos obtained. Both groups had a similar number of embryos transferred (42).

Interestingly, a study involving young women undergoing fertility preservation (dual triggering group: 30.9 years vs. hCG-only group: 29.6 years) found no difference in the number of retrieved oocytes (5.3 ± 3.5 vs. 5.0 ± 2.7, *p* = 0.655). However, the number of mature oocytes was significantly higher in the dual triggering group (3.7 ± 2.7 vs. 2.3 ± 1.7, *p* = 0.010) (43).

The results of studies in patients with LOR are summarized in **Table 1**.

**Table 1:** Studies of dual triggering in patients with low response to ovarian stimulation.

Randomized controlled study/Systematic review and meta-analysis												
Author/Design	N	Criteria	Groups	No. of retrieved oocytes	N° MII	Fertilization rate	Total embryos	Good quality embryos	Transferred embryos	Cancellation	Clinical pregnancy	LBR
Maged AM et al 2020 (40) (RCT)	160	Low response	G1: 10–000 IU hCG + 0.2 mg triptorelin G2: 10–000 IU hCG	G1: 5.3 ± 1.9 G2: 4.5 ± 2.4, *p =* 0.014	G1: 3.8 ± 1.4 G2: 3.1 ± 1.7, *p =* 0.004	NI	G1: 2.7 ± 1.1 G2: 1.9 ± 1.2 *p =* 0.001	G1: 2.3 ± 1.0 G2: 1.1 ± 0.2 *p =* 0.021	G1: 2.2 ± 0.9 G2: 1.6 ± 0.9 *p =* 0.043	G1: 7.5% G2: 20% *p = 0.037*	G1: 22.5% G2: 8.8% *p =* 0.028	NI
Sloth A et al 2022 (16) Systematic review and meta-analysis	2474 (7 studies)	Low response	G1: dual triggering G2: hCG alone	–	–	–	–	–	–	NI	1.62-fold increase in clinical pregnancy rate in G1	2.65-fold increase in live birth rate in G1
Zhou C et al 2022 (74) (RCT)	510	Age >35 years old	G1: hCG 6000 UI G2: 0,2 mg of Triptorelin G3: 0,2 mg of triptorelin + 2000 UI of hCG	G1: 3.6 ± 2.71 G2:3.81 ± 3.88 G3: 4.08 ± 2.79	G1: 2.78 ± 2.10 *p =* 0.376 G2:3.15 ± 2.95 *p =* 0.061 G3:3.54 ± 2.51 *p =* 0.353	IVF: G1:81.8% G2: 78.9% G3:76,7% ICSI: G1:82% G2:68% G3:74%	G1: 1.56 ± 1.66 *p =* 0.277 G2:1.45 ± 1.75 *p =* 0.008 G3:2.19 ± 2.11 *p =* 0.001	G1:44.1% *p =* 0.964 G2:44.3% *p = <*0.001 G3:59.2% *p = <*0.001	NI	NI	FET G1:22.7% *p =* 0.426 G2:40% *p =* 0.184 G3:42.1% *p =* 0.932 Frozen embryo transfer: G1:39.7% *p =* 0.154 G2:28.4% *p =* 0.962 G3: 39.3% *p =* 0.143	FET G1:13.6% *p =* 0.726 G2:20% *p =* 0.082 G3:36.8% *p =* 0.462 Frozen embryo transfer: G1:27.9% *p =* 0.044 G2:14.1% *p =* 0.537 G3: 32.6% *p =* 0.007
He FF et al 2023 (72) Systematic review and meta-analysis	898 patients (7 studies)	Low response									2.20 fold increase in clinical pregnancy rate	
Retrospective cohort studies												
Chen K et al 2024 (73)	734	Poor ovarian reserve Bologna criteria	G1: rhCG 250 μg+ Triptorelin 0.2 mg G2: only rhCG 250 μg	G1: 3.60 ± 2.73 G2: 2.39 ± 1.90 *p =* <0.001	NI	G1: IVF: 73.9 ± 34.8 ICSI: 78.4 ± 34.8 G2: IVF: 70.8 ± 37.7 ICSI: 73.2 ± 42.2 IVF *p =*0.342 ICSI *p =*0.401	G1: 1.22 ± 1.13 G2: 0.95 ± 0.98 *p =*0.001	G1: 4 (2.2%) G2: 4 (3.1%)	G1: 1.22 ± 1.13 G2: 0.95 ± 0.98 *p = 0.001*	G1: 141/397 (35.5%) G2: 148/337 (43.9%) *p =*0.020	G1: 46/179 (25.70%) G2: 31/126 (24.6%) *p =*0.828	G1: 39/178 (21.9%) G2: 26/125 (20.8%) *p =*0.817
Mutlu I et al 2021 (15)	1010	Low response	G1: dual trigger: rhCG 250 μg + leuprolide acetate 0.2 mg G2: only rhCG 250 μg	G1:4.5 ± 2.4 G2: 3.1 ± 2.3 *p =* <0.001	G1: 3.4 ± 2.0 G2: 2.3 ± 1.9 *p =* <0.001	G1: 73.6% G2: 69.6% *p* = 0.009	NI	G1: 1.73 ± 0.62 G2: 1.55 ± 0.63 *p =* <0.001	G1: 1.75 ± 0.58 G2: 1.57 ± 0.60 *p* = <0.001	G1: 197 (29.2) G2: 213 (35) *p* = 0.03	G1: (per cycle) 131 (19.4) G2: (per cycle) 79 (13) *p* = 0.002	G1: 103 (15.3) G2: 59 (9.7) *p =* 0.003
Tulek F et al 2022 (14)	2999	Low response Poseidon 3/4	G1: triptorelin acetate 0,2 mg + rhCG 250 μg G2: rhCG 250 μg	G1: 4.11 ± 1.89 G2: 3.73 ± 1.60 *p =* 0.001	G1: 3.3 ± 1.67 G2:2.63 ± 1.25 *p* = 0.001	G1: 0.77 ± 0.29 G2: 0.72 ± 0.27 *p* = 0.001	G1: 2.34 ± 1.42 G2: 1.72 ± 0.81 *p* = 0.001	G1:1332 (78.3%) G2: 1945 (66.3%) *p* = 0.001	G1:1.42 ± 0.49 G2: 1.47 ± 0.50 *p* = 0.001	G1: 48 (4.5%) G2: 102 (5.3%) *p* = 0.001	G1: 309 (28.9%) G2: 483 (25%) *p* = 0.020	G1: 266 (24.9%) G2: 351 (18.2%) *p* = < 0.001
Chern CU et al 2020 (41)	384	Low response Poseidon 4	G1: rhCG 250 μg + GnRH agonist (lupro 2mg) G2: rhCG 250 μg	G1: 3.3 ± 2.7 G2: 1.6 ± 1.5, *p <*0.001	G1: 2.6 ± 2.0 G2: 1.3 ± 1.0, *p <*0.001	G1: 74.3 ± 37.4 G2: 77.8 ± 39.4 *p* = 0.440	G1: 2.2 ± 1.9 G2: 1.2 ± 1.0, *p <*0.001	G1: 0.9 ± 1.3 G2: 0.2 ± 0.5, *p <*0.001	G1: 2.1 ± 1.0 G2: 1.4 ± 0.8, *p <*0.001	NS	G1: 3.1% vs G2: 8.7%, *p* = 0.004	G1: 17.5% G2: 5.4%, *p=* 0.006
Kim SJ. et al 2020 (43)	122	Low response Oocyte preservation (Age <35 years and AMH <1.2ng/mL)	G1: 0.2mg triptorelin + rhCG 250 μg G2: rhCG 250 μg	G1: 5.3 ± 3.5 G2: 5.0 ± 2.7 *p* = 0.655	G1. 3.7 ± 2.7 (68.5%) G2: 2.3 ± 1.7 (45.6%) *P* = 0.010	NI	NI	NI	NI	NI	NI	NI
Lin MH et al 2019 (42)	427	Low response	G1: 0.2 mg triptorelin + hCG 6500 IU G2: hCG 6500 IU	G1: 3.27 ± 1.53 G2: 3.40 ± 1.36 *p =* NS	G1: 2.75 ± 1.42 G2: 2.85 ± 1.33 *p =* NS	G1: 73.1% G2: 58.6% *p =* 0.015	NI	NI	G1: 2.06 ± 1.11 G2: 1.67 ± 1.10 *p* = NS	G1: 6.1% (18/297) G2: 15.4% (20/130) *p <*0.003	G1: 33.0% (92/297) per cycle G2: 20.7% per cycle (27/130) *p* = NS	G1: 27.2% (76/297) per cycle G2: 13.1% (17/130) per cycle *p* =0.014

NI, no information; RCT, randomized controlled trial; NS, no statistically significant differences; N° MII, number of metaphase II oocytes; LBR, Live birth rate, G1, Group 1, G2, Group 2; rhCG, recombinant hCG; μg, micrograms; FET, fresh embryo transfer.

### High proportion of immature oocytes retrieved

Although there is no uniform criterion in the literature, it is generally accepted that up to 25% of oocytes in a cohort may be immature (metaphase I and/or germinal vesicle) following the administration of a final ovulation trigger (44).

Studies conducted on patients with a high percentage of immature oocytes have shown improvements in the number of retrieved oocytes and the proportion of mature oocytes after dual triggering. A retrospective cohort study involving 137 IVF cycles investigated whether, in the same patient with a prior cycle triggered with hCG, dual triggering improved the rates of mature oocytes. This study confirmed that in patients with more than 70% of retrieved immature oocytes in a previous cycle, dual triggering resulted in a statistically significant increase of up to 20% more mature oocytes, although live birth rates per stimulation were not evaluated (45). This improvement may be attributed to the additional exposure to LH and FSH provided by aGnRH, which mimics more physiological hormonal conditions.

These findings align with another study that evaluated the percentage of mature oocytes retrieved and the fertilization rate in a second IVF attempt using dual triggering. Patients with a history of more than 25% immature oocytes retrieved in a failed prior cycle with hCG received dual triggering. In this group, the total number of retrieved oocytes (11 vs. 9), the fertilization rate (83.3% vs. 66.7%), and the percentage of mature oocytes (75% vs. 38.5%) were significantly higher compared to the prior hCG-triggered cycle. Furthermore, the implantation, clinical pregnancy, and live birth rates per embryo transfer (ET) for the dual trigger group were 11.8% (7 of 59), 26.1% (6 of 23), and 17.4% (4 of 23), respectively (46).

Studies by Herbemont et al. (47) and Fabris et al. (48) reported even higher clinical pregnancy rates in women with dual triggering (46.8% vs. 27.6% and 43.6% vs. 26.9%, respectively). However, live birth rates were not provided. Variability in sample sizes and the proportion of immature oocytes in these studies could account for the observed differences in outcomes. In any case, the improvement in reproductive outcomes is associated with an increase in the number of mature oocytes and the cumulative clinical pregnancy rate. However, no studies have demonstrated an improvement in embryo quality with dual triggering. A systematic review and meta-analysis suggested improvement in endometrial receptivity after using dual triggering in fresh embryo transfer (49).

Summarizing the results of studies, dual triggering appears to increase the mean number of mature oocytes retrieved by approximately 3.4 (19.4%) in patients with prior cycles involving a high percentage of immature oocytes. However, the impact on live birth rates remains unclear due to heterogeneity in study designs and incomplete reporting of outcomes. Only five studies with a limited number of patients have explored this issue, of which four were retrospective cohort studies and one was a randomized controlled trial. The results of studies in patients with a high proportion of immature oocytes are summarized in **Table 2**.

**Table 2:** Studies of dual triggering in patients with a high proportion of immature oocytes.

Randomized controlled trials										
Author/Design	N	Criteria (% of inmature oocytes retrieved)	Groups	No. of oocytes retrieved	N° MII	Fertilization rate	Good quality embryos	Transferred embryos	Clinical pregnancy rate	LBR
Yan MH et al 2023 (17)	73	>50%	G1: 0.2 mg GnRH-a and r-hCG (6,500 IU), 40 and 34 h prior to OPU G2: hCG (6,500 IU)	G1: 17 (12.5) G2: 15 (11.8) *p=*0.088	G1: 15 (9.0) G2: 7.50 (4.0) *p =*<*0.001*	G1: 75 (33.5) G2: 65.0 (39.5) *p=*0.032	G1: 4.0 (4.50) G2: 1.0 (3.75) *p=*<0.001	NI	G1: (cumulative rate) 25/36 (69.4) G2: (cumulative rate) 10/25 (40.0) *p=*0.035	G1: (cumulative rate) 24/36 (66.7) G2:9/25 (36.0) *p=*0.022
Retrospective cohort studies										
Ben-Haroush A et al., 2020 (45)	137	≥25%	G1: dual trigger G2: hCG Not specify dose.	G1: 10.3 ± 6.2 G2: 8.9 ± 6.1 *p=*0.011	G1:81 ± 18 *G2:*81± 18 *p=*0.997	G1: 58 ± 24 G2:53 ± 35 *p=*0.011	NI	NI	NI	NI
Herbemont C et al 2019 (47)	47	≥25%	G1: standard dose hCG + 0.2mg of triptorelin G2: hCG	G1: 12.9 ± 6.4 G2: 11.7 ± 5.9	G1:9.1 ± 5.9 G2: 5.8 ± 4.4 *p=0,0003*	G1: 64.9 ± 24.5 G2: 56.7 ± 31.1 *p=NS*	G1: 2.3 ± 2.7 G2:1.5 ± 1.7 *p= 0.03*	NI	G1:46.8% G2: 27.6% *p =* 0.05	NI
Fabris AM et al 2017 (48)	NI	> 50%	G1: hCG + aGnRH G2: hCG Unspecified doses	G1: 7.0 ± 4.4 G2: 5.5 ± 2.7 *p=0,02*	G1:5.3 ± 3.6 G2: 2.4 ± 2.2 *p=<0.001*	G1:82.5 ± 32.8 G2:79.6 ± 53 *p=0.7*	NI	NI	G1:43.6% G2:26.9%	NI
Griffin D et al 2014 (46)	54	> 25%	G1:1mg of GnRH-a + hCG 5000 G2: 10000 UI hCG	G1:11 G2: 9 *p = 0.02*	G1: 7 G2:3 *p <0.001*	G1:66.7% (40-100) G2:83.3% (72.4-93.8) *p = NS*	NI	G1:2 G:2: 1 *p = NS*	26.1%	17.4%

NI, no information; NS, no statistically significant differences; MII, mature oocytes; LBR, Live birth rate, G1, Group 1; G2, Group 2.

### Low fertilization rate

A low fertilization rate is defined as fertilization of fewer than 65% of oocytes during intracytoplasmic sperm injection (ICSI) or fewer than 60% during conventional IVF (50). The incidence of total fertilization failure is around 3% after ICSI with normozoospermic samples and ranges from 5% to 20%, averaging 10%, after conventional IVF (51, 52).

Two retrospective cohort studies have examined the use of dual triggering to address low fertilization rates. In one study, dual triggering significantly improved fertilization rates in ICSI cycles compared to prior cycles with hCG-only triggering (53). Another study showed improved fertilization rates (up to 16.4%) with dual triggering, as well as higher ongoing pregnancy rates (27.5% vs. 5.67%) and live birth rates per ICSI-ET cycle (20.2% vs. 3.46%) compared to the hCG group (54). The results of studies in patients with low fertilization rates are summarized in **Table 3**.

**Table 3:** Studies of dual triggering in patients with a low fertilization rate.

Retrospective cohort studies										
Author/Design	N	Criteria	Groups	No. of oocytes retrieved	N° MII	Fertilization rate	Transferred embryos	Positive βhCG	Clinical pregnancy rate	LBR
Elias RT et al 2017 (54)	427	Fertilization <20% in two ICSI cycles	G1: 10000UI hCG G2: 4mg GnRHa + 10000UI hCG	G1: 9 (5-14) G2: 10 (5-13) *p =* 0.56	G1: 69.8% G2: 82.1% *p = 0.03*	G1: 17.9 ± 3.61% G2: 42.1 ± 10.8% *p <*0.001	G1: 2.51 (± 0.89) G2: 2.67 (± 0.64) *p =* 0.08	G1: 10.7% G2: 18.1% *p=*0.13	G1: 18 (5.67%) G2: 30 (27.5%) *p <* 0.001	G1: 11 (3.46%) G2: 22 (20.2%) *p<*0.001
Pereira N et al., 2016 (53)	156	Fertilization <40% in an ICSI cycle	G1: hCG according to E2 values G2: 2mg of GnRHa + 1500IU hCG	G1: 13 (9-16) G2: 13 (8-17) *p =* NS	G1: 70.2% G2: 84.2% *p = 0.02*	G1: 35.3% G2: 59.2% *p < 0.01*	G1: 1.69 (± 0.35) G2: 1.72 (± 0.29) *p =* NS	G1: 31.2% G2: 42.9% *p=* NS	G1: 40.4% G2: 54.5% P = 0.03	G1: 33.3% G2: 45.5% *p =*0.03

NS, no statistically significant differences; MII, metaphase II oocytes; LBR, Live birth rate, G1, Group 1; G2, Group 2.

Even though it is not among the recognized indications, dual triggering has been studied in other patient groups, such as normo-responsive individuals and even those with high ovarian response to stimulation.

### Normo-responsive patients

Studies on normo-responsive patients undergoing ovarian stimulation can be categorized into two groups based on study design.

In the first group, RCTs were conducted. In these patients, the indications for dual triggering varied. One prospective randomized study included 155 patients with normal ovarian reserve (anti-Müllerian hormone [AMH] > 1 ng/mL; antral follicle count [AFC]: 6–20). Patients were randomized to receive either hCG (78 patients) or dual triggering (77 patients). The primary outcome was the number of mature oocytes and high-quality embryos. Results showed statistically superior outcomes in the dual triggering group for all variables studied: retrieved oocytes (13.4 vs. 11.1, *p* = 0.002), mature oocytes (10.3 vs. 8.6, *p* = 0.009), mean number of blastocysts (3.9 vs. 2.9, *p* = 0.01), and good-quality embryos (2.4 vs. 1.4, *p* = 0.001). No differences in clinical pregnancy rates were found between groups (55).

Another randomized study involving 192 patients with normal ovarian reserve showed a higher number of retrieved oocytes (10.85 ± 4.71 vs. 9.35 ± 4.35, p = 0.009) and total embryos (6.86 ± 4.16 vs. 5.34 ± 3.80, *p* = 0.007) in the dual triggering group. However, there were no significant differences in the number of mature oocytes, implantation rates, or clinical pregnancy rates. Live birth rates (LBR) were not reported (56). A separate study found a higher proportion of good-quality embryos (73.8% vs. 47.5%, *p* = 0.001) in the dual triggering group (57).

It is important to note that many studies assessing ovarian stimulation compare only the first embryo transfer. Since the best embryos are usually selected for the initial transfer, these comparisons often reflect differences between the best embryos in each cohort rather than the overall contribution of stimulation. Ideally, cumulative pregnancy rates should be evaluated until the first child is born.

A smaller randomized study did not find significant differences in reproductive outcomes (58). In this group of patients, none of the studies reported an increased risk of ovarian hyperstimulation syndrome (OHSS) with dual triggering compared to hCG. The results of randomized studies in normo-responsive patients are summarized in **Table 4**.

**Table 4:** Studies of dual triggering in patients with normal response to ovarian stimulation.

Randomized controlled trials/Systematic Review and Metanalysis														
Author/Design	N	Patients	Groups	No. of oocytes retrieved	N° MII		Fertilization rate	Total embryos	Good quality embryos	Transferred embryos	OHSS	Clinical pregnancy	LBR	
He FF et al 2023 (72) Systematic review and meta-analysis	898 patients (7 studies)	Normal response			1.34 fold increase				1.14 fold increase			1.37 fold increase in clinical pregnancy rate		
Hass J et al 2020 (55)	155	Normal response (AMH:> 1ng/mL, AFC: 6-20)	G1: hCG 10000 IU G2: 0.5mg GnRHa plus 10000 IU hCG	G1: 11.1 G2: 13.4 *p* = 0.002	G1: 8.6 G2: 10.3 *p=*0.009		G1: 6.3 G2: 7.8 *p=*0.007	G1: 2.9 G2: 3.9 *p=*0.01	G1: 1.4 G2: 2.4 *p=*0.001	G1: 45% G2: 65% *p=*0.003	There was no OHSS in any of the groups	G1: 51.5% G2: 52.1% *p=*1	G1: 32% G2: 45% (cumulative per patient) *p=*0.11	
Eftekhar M et al., 2017 (56)	192	Normal response (AMH:> 1ng/ml, AFC: 3-15)	G1: 6500IU hCG G2: 6500 IU hCG plus 0.2 mg GnRHa	G1: 9.35 ± 4.35 G2: 10.85 ± 4.71 *p=* 0.009	G1: 7.98 ± 3.85 G2: 8.80 ± 3.99 *P=* 0.12		NI	G1: 5.34 ± 3.80 G2: 6.86 ± 4.16 *p=* 0.007	NI	G1: 1.66 ± 0.82 G2: 1.72 ± 0.86 *p=* 0.61	NI	G1: 22.6% G2: 26.3% *p=* 0.30	NI	
Mahajan N et al., 2016 (58)	76	Normal response (AMH: <4ng/ml, AFC: <12)	G1: 1mg luperide plus 5000IU hCG G2: 10000 IU hCG.	G1: 10 ± 5,6 G2: 8.7 ± 5 *p=* 0.2816	G1: 8.4 ± 5 G2: 7.2 ± 4 *p=* 0.2588		G1: 5.9 ± 4.2 G2: 5.6 ± 3.3 *p=* 0.7390	G1: 4.0 ± 3.0 G2: 4.0 ± 2.4 *p =* 0.8991	NI	NI	NI	NI	NI	
Decleer W et al 2014 (57)	120	Normal response	G1: 5000IU hCG G2: 0.2mg of triptorelin + 5000 IU hCG	NI	G1: 9.2 ± 6.7 G2: 10.3 ± 6.8 *p =* NS		G1: 34% G2: 22% *p =*NS	G1: 1.5 ± 2.9 G2: 2.2 ± 2.9 *p* = NS	G1: 28/59 (47.5%) G2: 45/61 (73.8%) *p =* 0.001	NI	There was no OHSS in any of the groups	G1: 26/59 (44.1%) G2: 19/61 (31.1%) *p =* NS	NI	
Retrospective cohort studies														
Author/Design	N	Patients	Groups	No. of oocytes retrieved		N° MII	Fertilization rate	Total embryos	Good quality embryos	Transferred embryos	OHSS	Clinical pregnancy		LBR
Dong Li et al 2022 (63)	520	All patient undergoing ART	G1: 6500UI of hCG + 0.2mg triptorelin G2: hCG	G1: 7.04 ± 3.87 G2: 6.53 ± 3.36 *P=*0.455		NI	G1: 67.72 ± 25.63 G2: 65.54 ± 26.58 *P=*0.657	G1: 2.81 ± 1.85 G2: 2.84 ± 1.73 *P=*0.191	G1: 0.84 ± 0.98 G2: 0.79 ± 1.05 *P=* 0.782	G1: 1.75 ± 0.43 G2: 1.77 ± 0.42 *P=*0.828	G1: 1/57 (1.8) G2: 1/57 (1.8) *P=*1.0	G1: 27/57 (47.4) G2: 24/57 (42.1) *P=*0.572		G1: 15/57 (28.1) G2: 20/57 (33.3) *P=*0.542
Gao F et al 2021 (62)	469	Normal response	G1: 6500UI of hCG + 0.2mg triptorelin G2: hCG	G1: 11.24 ± 4.76 G2: 10.24 ± 4.27 *P=* 0.02		G1: 8.37 ± 4.44 G2: 7.67 ± 3.69 *P=* 0.07	G1: 84.40% ± 17.71 G2: 83.44% ± 22.20 *P=* 0.60	G1: 7.37 ± 3.69 G2: 6.62 ± 3.26 *P=* 0.02	G1: 1.53 ± 1.53 G2: 1.31 ± 1.40 *P=* 0.10	G1: 2.60 ± 1.22 G2: 2.61 ± 1.15 *P=* 0.97	NI	G1: 41.94% G2: 40.85% *P=* 0.89		G1: (cumulative rate): 54.07% G2: (cumulative rate): 59.30% *P=* 0.26
Şükür YE et al 2020 (59)	200	Normal response (6–14 oocytes)	G1: 0.2 mg triptorelin acetate G2: 0.2 mg triptorelin acetate plus 1500IU hCG G3: 10000IU hCG	G1: 7.4 ± 4.9 G2: 9.2 ± 5.3 G3: 7.6 ± 4.5 *p* = 0.087		G1: 6.2 ± 4.2 G2: 7.2 ± 4.7 G3: 5.6 ± 3.7 *p=* 0.095	G1: 69 ± 42% G2: 70 ± 33% G3: 62 ± 29% *p =* 0.500	NI	G1: 3.2 ± 2.9 G2: 4.4 ± 3.2 G3: 2.9 ± 2.1 *p=* 0.014	G1: 1.1 ± 0.7 G2: 1.2 ± 0.6 G3: 1.2 ± 0.5 *p =* 0.291	NI	G1: 13 (23.2%) G2: 20 (33.9%) G3: 26 (30.6%) *p=* 0.112		G1: 12 (21.4%) G2: 18 (30.5%) G3: 24 (28.2%) *p=* 0.126
Zhou X et al 2018 (60)	325	Normal response	G1: 5000-10000IU of hCG + 0.2mg triptorelin G2: hCG	G1: 9.64 ± 3.99 G2:9.13± 3.89 *p=* 0.719		G1: 6.65 ± 3.61 G2:5.88 ± 3.28 *p=0.154*	G1: IVF 62.1% ICSI:69.2% *p=* 0.119 G2: IVF:58.5% ICSI: 63.6% *p=* 0.173	G1:5.86 ± 2.97 G2:4.95 ± 2.35 *p=* 0.004	G1:2.81 ± 2.21 G2:2.29 ± 1.55 *p=* 0.011	G1: 1.93 ± 0.65 G2: 2.07 ± 0.41 *p=* 0.082	G1:0 G2: 1 *p=* 0.311	G1: 62.3% G2: 52.6% *p=* 0.225		G1: 54.3% G2:40.8% *p=* 0.083
Lin MH et al 2013 (61)	378	Normal response (3–20 oocytes, AMH:> 1ng/mL)	G1: 6500 IU hCG G2: 0.2mg triptorelin plus 6500 IU hCG	G1:10,10 ± 4,58 G2: 12,36 ± 6,64 *P <* 0.01		G1: 8.03 ± 4.51 G2: 10.53 ± 6.47 *p <* 0.01	NI	G1: 5.3 ± 3.6 G2: 5.8 ± 3.8 *p=* NS	G1: 2.9 ± 2.3 G2: 2.9 ± 3.1 *p=* NS	G1: 2.84 ± 0.85 G2: 2.79 ± 0.87 *p=* NS	NI	G1: 40.11% (75/187) G2: 50.79% (97/191) *p=* 0.047		G1: 30.49% (57/187) G2: 41.36% (79/191) *p=* 0.042

AFC, antral follicle count; NI, no information, NS, no statistically significant differences; OHSS, ovarian hyperstimulation syndrome; LBR, Live birth rate; G1, Group 1; G2, Group 2.

The second group of studies on normo-responsive women includes retrospective studies that compare patients with themselves. These studies aimed to assess improvements in clinical outcomes (e.g., number of mature oocytes, good-quality embryos, and live birth rates). Most studies failed to show significant improvements with dual triggering compared to hCG. However, one study involving 200 normo-responsive patients reported a significant improvement in the proportion of good-quality embryos but not in the number of retrieved metaphase II (MII) oocytes (59). Another study showed better fertilization rates and a higher number of embryos (60), while a third study with a larger sample size (n = 378) found improvements in the number of retrieved and mature oocytes (*p* < 0.01), as well as better clinical pregnancy and live birth rates (*p* = 0.047 and *p* = 0.042, respectively) with dual triggering (61).

Recent retrospective cohort studies support that dual triggering does not result in significant differences in cumulative LBR (54.07% vs. 59.30%) or clinical pregnancy rates compared to hCG alone in normo-responsive patients (62, 63). Overall, studies with weaker designs have not demonstrated clear benefits of dual triggering in terms of pregnancy rates or LBR for women with a normal response to ovarian stimulation. The results of non-randomized studies in normo-responsive patients are summarized in **Table 4**.

### High responders

Dual triggering has also been evaluated in women with high ovarian response to stimulation. However, there was no uniformity in the control group protocols in the analyzed studies, with variations in aGnRH doses (e.g., 0.2 mg of Decapeptyl, 4 mg of leuprolide acetate, and 1 mg of leuprolide acetate) and hCG doses (10,000 IU in most studies, with 8,000 IU in some cases) (64–66).

The largest study found significant differences in the number of retrieved oocytes (control group: 18.54 ± 4.38 vs. dual triggering: 21.63 ± 7.43) but reported similar fertilization rates between groups (66). The number of retrieved mature oocytes was not provided. Other studies showed similar results for MII oocyte numbers.

The risk of moderate OHSS was higher in patients triggered with dual triggering compared to conventional hCG triggering [hCG 10,000 IU: 32 (32.65%) vs. dual triggering: 17 (39.53%)], with a similar finding for severe OHSS [hCG 10,000 IU: 2 (2.04%) vs. dual triggering: 1 (2.34%)]. Three patients with severe OHSS required hospitalization and paracentesis (67).

The lack of uniformity in protocols and poor study designs make it impossible to draw definitive conclusions about the use of dual triggering in high responders. Currently, triggering with aGnRH alone is a safer strategy to prevent OHSS. The results of studies in high responders are summarized in **Table 5**.

**Table 5:** Studies of dual triggering in patients with high ovarian response.

Retrospective cohort studies												
Author/Design	N	Patients	Groups	No. of oocytes retrieved	N° MII	Fertilization rate	Total embryos	Good quality embryos	Transferred embryos	OHSS	Pregnancy BhCG+	LBR
Li S et al 2018 (67)	226	High response	G1 control: 10000IU hCG G2: 0.2mg GnRH + 2000IU hCG G3: 8000IU hCG	G1: 18.54 ± 4.38 G2: 21.63 ± 7.43 G3: 20.27 ± 5.42 G1 vs. G3*: p = 0.002* *p=* 0.034	NI	G1: 68.82 ± 19.49 G2: 64.86 ± 18.67 G3: 70.81 ± 17.10 *p=*NS	G1: 10.19 ± 4.61 G2: 13.58 ± 7.21 G3: 12.25 ± 5.28 G1 vs G3: *p=* 0.001 G1 vs G2: *p=* 0.011	G1: 52.83 ± 23.71 G2: 64.21 ± 22.34 G3: 59.60 ± 19.37 G1 vs G3: *p=* 0.005 G1 vs G2: *p=* 0.038	NI	G1: 56 (57.14%) G2: 17 (39.53%) G3: 37 (43.53%) In G3: 5 severe OHSS patients	NI	NI
Oliveira SA et al 2016 (64)	24	High response	G1: 0.2mg GnRHa + 2500IU hCG G2: 0.2mg GnRHa	G1:7.03± 3.12 G2: 4.67 ± 1.63 *p=*NS	G1:5.38 ± 3.82 G2:3.32 ± 1.24 *p=*NS	NI	G1: 4.02 ± 2.34 G2: 2.80 ± 1.43 *p=*NS	G1:2.12 ± 1.68 G2:1.93 ± 1.45 *p=*NS	G1: 1.45 ± 0.82 G2: 1.53 ± 0.93 *p=*NS	NI	G1: 50.00% G2: 27.27% *p=*NS	G1: 44.4% G2: 16.13% *p=NS* per embryo transfer
O’Neill KE et al., 2016 (65)	177	High response	G1: 4mg GnRHa G2: 4mg GnRHa + 1000 IU hCG.	G1: 16.5 (11-21.5) 3 patients puncture without oocytes. G2: 17.5 (12–24)	G1: 70 (56–85) G2: 82 (74–91) *p<* 0.01	NI	NI	NI	G1: 45% G2: 88% *p <*0.01	G1: 0 G2:6 *p <*0.01	G1: 63% G2: 44% *p=* 0.12	NI
Griffin D et al., 2012 (66)	102	High response	G1: 1mg GnRHa G2: 1000 IU hCG + 1mg GnRHa	G1: 24 ± 10 G2: 23 ± 10 *p=*NS	NI	G1:81.9 ± 18.1 G2: 79.2 ± 13.9 *p= NS*	G1: 4.3 ± 4.7 G2: 3.6 ± 3.1 *p=* 0.03	NI	G1: 1.8 ± 0.4 G2: 1.8 ± 0.5 *p=* NS	NI	G1: 36.8% G2: 58.8% *p=* 0.03	G1: 30.9% G2: 52.9% *p=*0.03

NI, no information; NS, no statistically significant differences; OHSS, ovarian hyperstimulation syndrome; MII, metaphase II oocytes; LBR, Live birth rate; G1, Group 1; G2, Group 2.

### Empty follicle syndrome

EFS is characterized by the absence of oocyte retrieval from ovarian follicles that exhibit normal growth and estradiol levels (150–200 pg/mL per mature oocyte) at the time of triggering. Most cases are due to human error in administering hCG or aGnRH. However, other etiologies, such as partial hypothalamic disorders or profound pituitary suppression, may also result in EFS in cycles triggered by aGnRH.

The prevalence of EFS is estimated at 0.04–3.4% and increases with age. The primary purpose of ovulation triggers is to ensure adequate LH exposure for ovulation while inducing final oocyte maturation in multiple follicles. This allows most oocytes to be mature and ready for recovery 35–37 hours after bolus application. Inadequate LH exposure leads to insufficient maturation and EFS. For such patients, dual triggering may be a viable option, though literature on this indication is limited (68).

## Discussion

Dual triggering combines the strengths of hCG and aGnRH. This approach leverages hCG’s sustained LH activity and the ability of the aGnRH to induce an FSH surge. Its primary advantage is its ability to simulate the physiology of natural ovulation while providing a stronger local LH effect on the follicle, ensuring oocyte maturation. Additionally, it offers prolonged luteal support due to the strong LH effect of hCG on the follicle.

Most trials are retrospective and compare the use of dual triggering with conventional hCG induction. Few studies are randomized or systematic reviews.

Although the study designs are not robust enough to draw definitive clinical conclusions, dual triggering appears beneficial for patients with LOR and those with a high percentage of immature oocytes, where the total and mature oocyte counts increase. However, it remains unclear whether reproductive outcomes are significantly improved with this strategy.

The first systematic review and meta-analysis comparing hCG to dual triggering reported better reproductive outcomes in the dual triggering group. Interestingly, this improvement occurred despite no differences in primary outcomes, such as the number of retrieved oocytes, mature oocytes, or fertilized oocytes. Furthermore, implantation rates were similar, leaving the mechanism behind the better reproductive outcomes unclear (69).

In contrast, a more recent study with the same design concluded that the dual triggering protocol appears more effective in GnRH antagonist cycles, improving both embryo and pregnancy outcomes. This study also found that dual triggering was favored in terms of the number of retrieved oocytes and live birth rates (70). Another systematic review and meta-analysis evaluating various final oocyte maturation methods, including dual triggering, aGnRH, and FSH, compared to hCG alone, found no difference in pregnancy rates between the dual triggering and hCG groups. However, a statistically significant increase in the number of mature oocytes was observed in the dual triggering group (71).

The most recent systematic review and meta-analysis (72) yielded contradictory results in patients with normal ovarian response, suggesting a possible improvement in oocyte maturity and embryo quality.

Even the latest retrospective study supports the notion that dual triggering significantly increases the number of oocytes retrieved in patients with diminished ovarian reserve but has no effect on implantation rates, clinical pregnancy rates, live birth rates in fresh cycles, or cumulative live birth rates (73).

On the other hand, there is insufficient evidence to determine which trigger achieves the best outcomes in IVF patients aged >35 years. A recent randomized controlled trial (74) divided patients into three groups: hCG alone, aGnRH alone, and dual triggering. The number of retrieved oocytes in the dual trigger group was comparable to those in the hCG group and the aGnRH group. However, the numbers of good-quality embryos and viable embryos were significantly higher in the dual triggering group than in the hCG and aGnRH groups. The pregnancy rates after fresh embryo transfer in the dual trigger group were not superior to those in the hCG group. This suggests that women over 35 do not seem to be benefited of dual triggering.

It is important to note that this strategy affects both the oocyte and the endometrium. The effect of dual trigger on the endometrium in fresh embryo transfer has not been properly studied.

## Conclusions

Dual triggering combines hCG with an aGnRH to induce final oocyte maturation in IVF. The heterogeneity in protocols, inclusion criteria, study aims, and designs complicates the evaluation of dual triggering’s clinical benefits. Patients with a low response to ovarian stimulation may benefit from dual triggering, as it increases the number of retrieved oocytes and potentially improves reproductive outcomes compared to conventional hCG triggers. Similarly, in patients with previous cycles involving >25% immature oocytes, dual triggering significantly increased the number of retrieved oocytes and pregnancy rates. However, in patients with normal or high ovarian responsiveness, no differences were observed between conventional hCG and dual triggering.

Further robust studies are needed to clarify the clinical applications of dual triggering. Until such evidence is available, this strategy should remain part of research protocols rather than routine clinical practice.
